# Nicotine inhibits activation of microglial proton currents via interactions with α7 acetylcholine receptors

**DOI:** 10.1007/s12576-016-0460-5

**Published:** 2016-06-02

**Authors:** Mami Noda, AI Kobayashi

**Affiliations:** Laboratory of Pathophysiology, Graduate School of Pharmaceutical Sciences, Kyushu University, 3-1-1 Maidashi, Higashi-ku, Fukuoka, 812-8582 Japan

**Keywords:** Microglia, Nicotine, α7 nAChRs, Lipopolysaccharide, NADPH oxidase, Proton currents

## Abstract

Alpha 7 subunits of nicotinic acetylcholine receptors (nAChRs) are expressed in microglia and are involved in the suppression of neuroinflammation. Over the past decade, many reports show beneficial effects of nicotine, though little is known about the mechanism. Here we show that nicotine inhibits lipopolysaccharide (LPS)-induced proton (H^+^) currents and morphological change by using primary cultured microglia. The H^+^ channel currents were measured by whole-cell patch clamp method under voltage-clamp condition. Increased H^+^ current in activated microglia was attenuated by blocking NADPH oxidase. The inhibitory effect of nicotine was due to the activation of α7 nAChR, not a direct action on the H^+^ channels, because the effects of nicotine was cancelled by α7 nAChR antagonists. Neurotoxic effect of LPS-activated microglia due to inflammatory cytokines was also attenuated by pre-treatment of microglia with nicotine. These results suggest that α7 nAChRs in microglia may be a therapeutic target in neuroinflammatory diseases.

## Introduction

Nicotine, like several other abused drugs, is known to act on the reward system in the brain. It was shown that cues related to smoking induce not only a subjective emotional alteration but also sympathetic activation in smokers [1]. Apart from the reward system, according to epidemiological studies since the early 1990s, smoking lowers the risk of neurodegenerative diseases such as Alzheimer’s disease (AD) and Parkinson’s disease (PD) [2–4]. A marked decrease of nicotine receptor expression has been reported in AD patients [5, 6], PD patients [7, 8], and other neurodegenerative disease patients [9–13] including aging and dementia [14], suggesting the importance of the nicotinic receptors in brain function. Chronic nicotine infusion increases the basal level of acetylcholine (ACh) release in the cerebral cortex and enhances responses of cortical ACh release, but not in aged animals [15]. The lack of an effect of chronic nicotine in aged animals may be due to a decrease in nAChRs in the cerebral cortex during aging as mentioned above.

In the brain, glial cells are considered to be the principal pathologic response element; both microglial cells [16–18] and astrocytes [19]. Microglia are the primary immune cells in the central nervous system (CNS) (see review [20]). Under pathological conditions such as ischemia, trauma, and stroke, they are rapidly activated, and secrete various cytokines, including tumor necrosis factor (TNF)-α and interleukin-1β [17, 21]. Even minor pathology, such as early-life stress, changes in microglial function, such as increased motility, in adulthood [22]. Alternatively, microglia can exert neuroprotective functions by secreting growth factors or diffusible anti-inflammatory mediator (see review [20]). Over the past decade, many reports show beneficial effects of nicotine (see review, [23, 24]), but there are few electrophysiological analysis on microglia to explain the effect.

Voltage-gated H^+^ channels in all cells enable recovery from an acute acid load, though their expression is mainly restricted to immune cells [25]. H^+^ channels enable NADPH oxidase function by compensating cellular loss of electrons with protons. It is known that NADPH oxidase-mediated brain damage in stroke can be inhibited by the suppression of H^+^ channels [26]. It is also known that mouse and human brain microglia, but not neurons or astrocytes, expressed large H^+^ channels-mediated currents, and H^+^ channels were required for NADPH oxidase-dependent ROS generation in brain microglia in situ and in vivo [26]. Slowly activating outward H^+^ currents were measured in microglia during membrane depolarization [27–29]. Under pathological conditions such as neurodegeneration, pH homeostasis is reduced but H^+^ channels contribute to its recovery [30]. It was suggested that intracellular acidosis plays an important role in the progression of AD [31]. Production of ROS in neutrophil was decreased in H^+^ channels-deficient mice [32, 33]. Since H^+^ channels are crucial for oxidative stress-related brain disorders, microglial H^+^ channels might be one of the targets for nicotine. We therefore sought to examine the effect of nicotine on microglial H^+^ channels and to investigate its potential role in neuroprotection in inflammatory neuronal damage.

## Materials and methods

### Microglial cells

Microglial cells were isolated from the mixed cultures of cerebrocortical cells from postnatal day 1–2 C57BL/6 mice, as reported previously [34–36]. In brief, cortical tissue was trypsinized for 2 min, dissociated with a fire-polished pipette. Mixed glial cells were cultured for 9–12 days in Dulbecco’s modified Eagle’s medium (DMEM; Nissui) supplemented with 10 % fetal bovine serum (FBS; Hyclone Laboratories, Inc), 2 mM l-glutamine, 0.2 % d-glucose, 5 μg/ml insulin, 0.37 % NaHCO_3,_ 100 U/ml penicillin, 100 μg/ml streptomycin, with medium changes every 5 day. Microglial cells were then separated from the underlying astrocytic layer by gentle shaking of the flask for 2 h at 37 °C in a shaker-incubator (120 rpm). Microglial cells were then isolated as strongly adhering cells after unattached cells were removed. The purity of microglia was >98 %, which was evaluated by staining with Iba-1, a marker for microglia/macrophage.

### Electrophysiological measurements

Whole-cell recordings of microglial cells were made as reported previously [34, 37] using an Axopatch-200B amplifier (Axon Instruments), under voltage-clamp conditions at holding potential of −60 mV. The voltage pulses of 1 s were applied from −100 to +100 mV with a 20-mV interval. The proton currents were measured according to previous reports [28, 38, 39] using a patch pipette containing (in mM): 2-morpholinoethanesulfonic acid [40], 120; NMDG aspartate, 85; BAPTA, 1; MgCl_2_, 3. The pH was adjusted to 5.5 with 1 N CsOH. The pipette resistance was 6–9 MΩ. The external solution contained (in mM): NMDG-aspartate, 85; HEPES, 100; CaCl_2_, 1; MgCl_2_, 1. The pH was adjusted to 7.3 with 1 N CsOH. The osmolarity was ~310 mOsm. The temperature monitored in the recording dishes was 37 °C.

### Drugs

The NADPH oxidase inhibitor diphenyleneiodonium (DPI; SIGMA) was dissolved in DMSO at 10 mM, and the solutions were diluted into the control medium to prepare working solution (1 μM). The concentration of DMSO in the medium was 0.01 %. As control for DPI application, the same amount of DMSO (0.01 %) was added as vehicle. (−)-Nicotine hydrogen tartrate salt, α-bungarotoxin-tetramethylrhodamine (α-Bgt) and methyllycaconitine (MLA) citrate salt hydrate were purchased from SIGMA.

### Immunocytochemistry

Cultured mouse microglial cells were stained according to previous reports [41, 42]. Briefly, murine microglial cells seeded on the slide glass (4 × 10^4^ cells/dish) were fixed with 4 % paraformaldehyde (PFA), then initially rinsed three times before treated with a primary antibody against microglia (rabbit anti-mouse Iba-1, Wako Pure Chemical Industries, Osaka, Japan, 1:2000 in 10 % block ace) overnight at 4 °C, and then incubated with the Alexa Fluor 488-conjugated goat anti-rabbit IgG (1:1000) for 3 h at 18 °C, Texas Red-conjugated phalloidin (2U/ml 11 % BSA) for 1 h at 18 °C. A series of images were examined with a confocal laser-scanning microscope (LSM510; Carl Zeiss, Oberkochen, Germany).

### Western blotting

Expression protein level of proton channel in cultured microglial cells was examined by Western blotting relative to β-actin. Cultured microglial cells (10^6^ cells for control and LPS or nicotine/LPS group, each) were plated and incubated for 24 h. After treatment of LPS (SIGMA) (1 μg/ml) for 24 h with or without pretreatment with nicotine (1 μM) for 1 h, the cells were lysed. The total lysates derived from culture microglia were resolved in 7.5 % sodium dodecyl sulfate-polyacrylamide gel electrophoresis and transferred to a polyvinylidene difluoride membrane. The membrane was blocked for 30 min in Tris-buffered saline containing 0.1 % Tween 20 (TBS-T) and 10 % non-fat dried milk. Then, the membranes were incubated with primary antibody (HVCN1 (K-11): sc-136712, Santa Cruz Biotechnology) (1:500) and anti-β-actin (1:1000, Sigma) in TBS-T containing 10 % non-fat dried milk), overnight, at 4 °C. After washing with TBS-T, the membrane was incubated with horseradish peroxidase-conjugated anti-rabbit IgG antibody (1:5000 in TBS-T containing 1 % non-fat dried milk) (Millipore) for 1 h at room temperature. The membrane was washed four times with 1 % non-fat dried milk and then with TBS-T. HVCN1 proteins were visualized using ECL plus Western blot detection system (GE Healthscience) and analyzed using an LAS-4000 imaging system.

### Collection of microglial conditioned medium (MCM)

Cultured microglial cells from C57/BL6 mice were plated on 24-well glass dishes and incubated with serum-free DMEM for 24 h according to a previous report [36]. Microglial cells were incubated with LPS (1 μg/ml) for 24 h. Nicotine (1 μM) was pre-treated for 1 h before application of LPS. To completely remove LPS and nicotine from the medium, the cultures were rinsed three times for 5 min with PBS, and then incubated in fresh serum-free DMEM for 24 h. The media was centrifuged (1200 × *g*, 10 min) and the supernatants were collected. These were supposed to include LPS-stimulated inflammatory cytokines from microglia, referred to as LPS-treated microglial conditioned medium (LPS–MCM). LPS–MCM was preserved at −30 °C until use.

### Neuronal cell culture from hippocampus and cortex

Hippocampal and cortex neurons were obtained from embryonic day 14–16 C57BL/6 mice as described previously [36, 43]. Briefly, neurons were cultured at 37 °C in a 5 % CO_2_/95 % O_2_ incubator for 5–7 days with neurobasal medium (GIBCO) containing 2 % B27 supplement (GIBCO) and 0.5 mM l-glutamine (GIBCO).

### Neuronal cell accounting

The number of live neuronal cells with or without drug application was checked by using a cell counting kit-8 (Dojindo, Kumamoto, Japan) according to the manufacturer’s instructions.

### Statistical analysis

The results were expressed as the mean ±SEM. The data were compared with Student’s* t* test or one-way ANOVA followed by Scheffé test using the software package StatView 5.0j. Values of *p* < 0.05 were considered statistically significant.

## Results

### Proton currents are increased in activated microglia

**Fig. 1:**
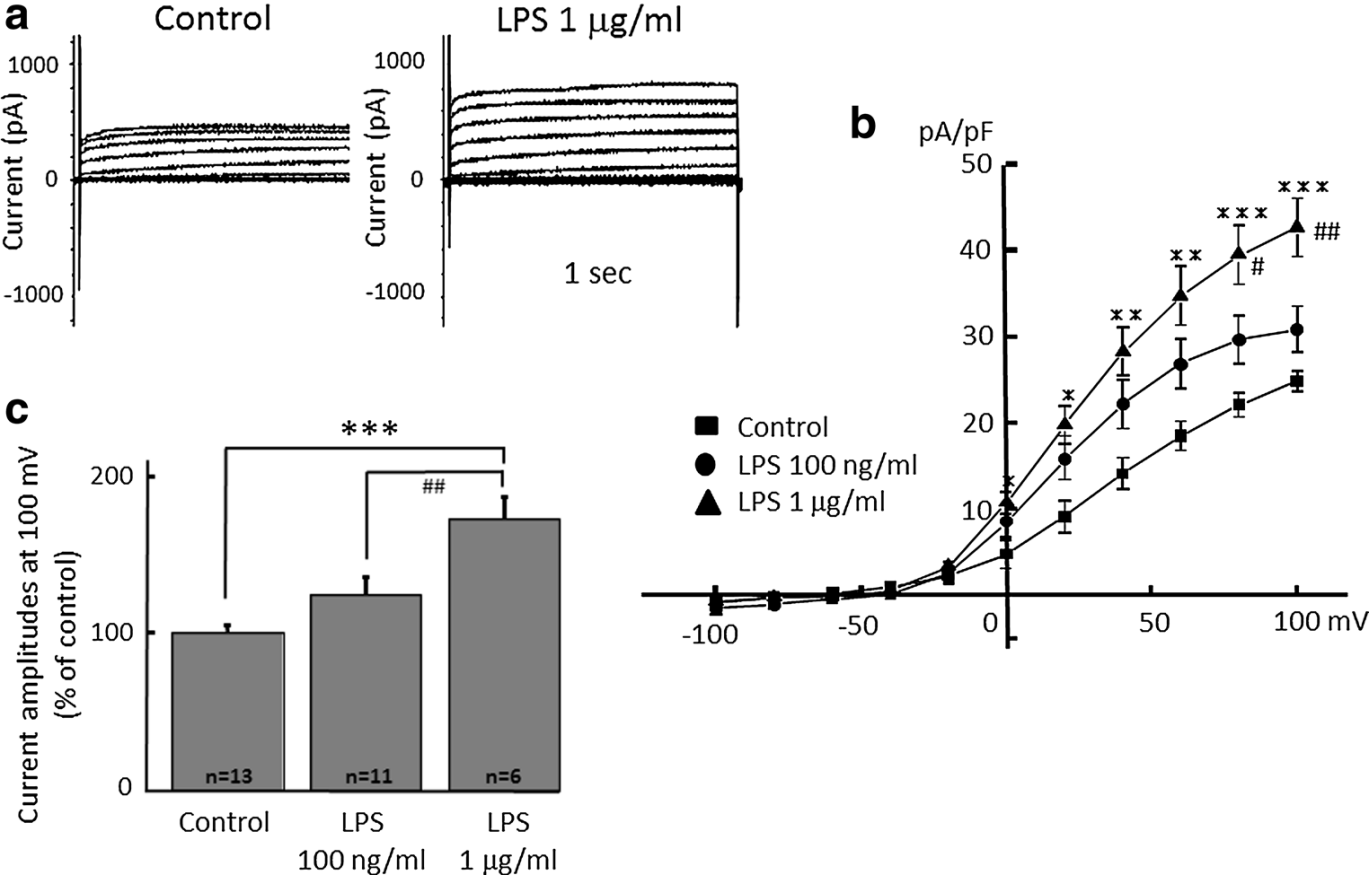
Microglial H^+^ currents are significantly increased by application of lipopolysaccharide (LPS) in a dose-dependent manner. **a** Current traces from −100 to +100 mV from the holding potential of −60 mV for 1 s with or without (control) application of LPS (1 μg/ml) for 24 h are shown. **b** Current–voltage (I–V) relationships in the absence of LPS (control, *filled square*), with 100 nM/ml (*filled circle*) and 1 μg/ml LPS (*filled upright triangle*) are shown. **c** The relative current amplitudes at +100 mV in **b** are shown. *p < 0.05, **p < 0.01, ***p < 0.005 compared to control.^#^ *p* < 0.5, ^##^ *p* < 0.01 compared to LPS (100 ng/ml)

H^+^ currents in microglia were changed in morphology and functional state by LPS [44]. First, to confirm that H^+^ currents are affected in activated microglia, cultured microglial cells were stimulated by Gram-negative bacterial LPS (Fig. 1). LPS (100 ng/ml and 1 μg/ml) was applied for 24 h and H^+^ currents were recorded by whole-cell patch clamp method at the holding potential of −60 mV. The H^+^ currents at positive potentials were increased after treatment of LPS (1 μg/ml) (Fig. 1a). The current–voltage relationships showed that 1 μg/ml, but not 100 ng/ml LPS, significantly increased the amplitude of H^+^ currents at the end of a 1-s pulse between +20 and +100 mV of membrane voltage (Fig. 1b). The current amplitudes at +100 mV showed that 1 μg/ml LPS showed significant increase compared to those in control and 100 ng/ml LPS, suggesting a concentration-dependent effect of LPS (Fig. 1c).

### Increased proton currents in activated microglia is attenuated by blocking NADPH oxidase

**Fig. 2:**
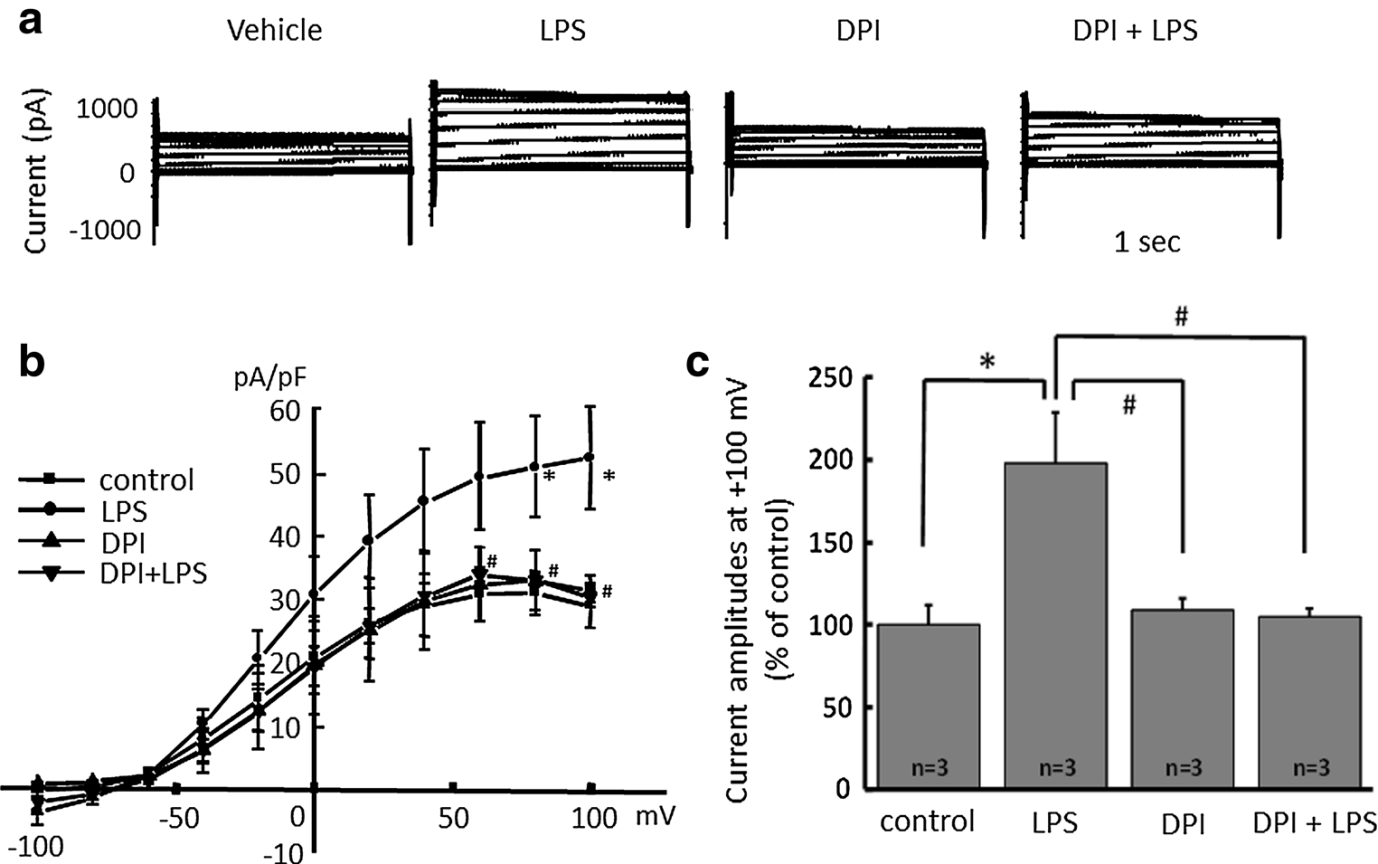
NADPH oxidase inhibitor attenuates LPS-induced microglial H^+^ currents. **a** Current traces from −100 to +100 mV from the holding potential of −60 mV for 1 s are shown. LPS (1 μg/ml) was applied for 24 h and NADPH oxidase inhibitor, dibenziodolium chloride (DPI, 1 μM), was pre-treated for 1 h prior to the application of LPS. **b** I–V relationships in the absence of LPS (control with vehicle, *filled square*), with 1 μg/ml LPS (*filled circle*), 1 μM DPI alone (*filled upright triangle*), and DPI + LPS (*filled downright triangle*) are shown. **c** The relative current amplitudes at +100 mV in **b** are shown. *p < 0.05 compared to control. ^#^ *p* < 0.5 compared to LPS (1 μg/ml)

It is known that LPS upregulates NADPH oxidase in neutrophils [45] and H^+^ channels and NADPH are important in phagocytes [30, 46]. To test whether or not NADPH oxidase is important for the LPS-induced H^+^ current, an inhibitor of NADPH oxidase, dibenziodolium chloride (DPI), was used. Pre-treatment with DPI (1 μM) for 1 h attenuated LPS (μg/ml)-induced H^+^ current (Fig. 2a). DPI alone did not have any effect. The current–voltage relationships showed that 1 μg/ml LPS significantly increased the amplitude of H^+^ currents at the end of a 1-s pulse between +60 and +100 mV of membrane voltage (Fig. 2b). The relative current amplitudes of H^+^ currents at +100 mV showed a significant increase by 1 μg/ml LPS, while pre-incubation with DPI almost completely inhibited LPS-induced H^+^ currents (Fig. 2c).

### Inhibition of LPS-induced proton currents and morphological change of microglia by nicotine

**Fig. 3:**
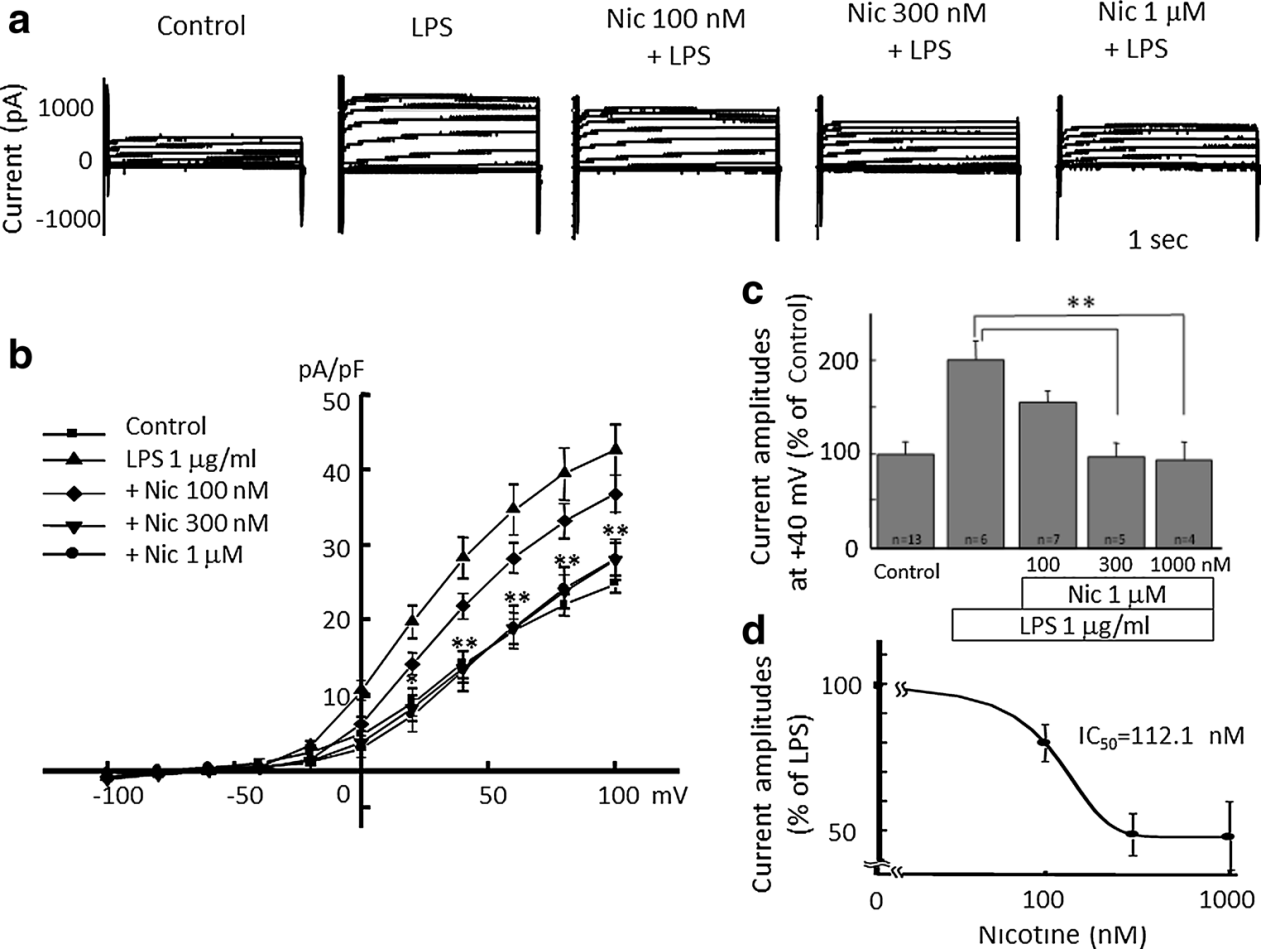
Nicotine dose-dependently attenuates LPS-induced microglial H^+^ currents. **a** Current traces from −100 to +100 mV from the holding potential of −60 mV for 1 s are shown. Application of LPS (1 μg/ml) was for 24 h and nicotine at concentration of 100 nM, 300 nM, and 1 μM were pre-treated for 1 h before application of LPS. **b** I–V relationships in the absence of LPS (control, *filled square*), with 1 μg/ml LPS (*filled upright triangle*), LPS with 100 nM (*open diamond*), 300 nM (*filled downright triangle*), and 1 μM Nic (*filled circle*) are shown. **c** The relative current amplitudes at +40 mV in **b** are shown. **d** Dose-dependent effect of Nic on LPS-induced microglial H^+^ currents is shown. The half inhibitory concentration (IC_50_) of Nic is 112.13 nM. **p < 0.01 compared to LPS (1 μg/ml)

Next, we tested whether or not LPS-induced H^+^ current is inhibited by nicotine. LPS-induced H^+^ currents were attenuated by pre-incubation of nicotine for 1 h in a dose-dependent manner (Fig. 3a). The current–voltage relationships for H^+^ current amplitudes at the end of a 1-s pulse recorded after LPS pretreatment were significantly reduced by 300 nM and 1 μM nicotine at positive membrane potential (Fig. 3b). The current amplitudes at +40 mV showed significant inhibition of LPS-induced H^+^ currents by 300 nM and 1 μM nicotine, but not with 100 nM nicotine (Fig. 3c). The half inhibitory concentration (IC_50_) of nicotine was 112.1 nM (Fig. 3d).

**Fig. 4:**
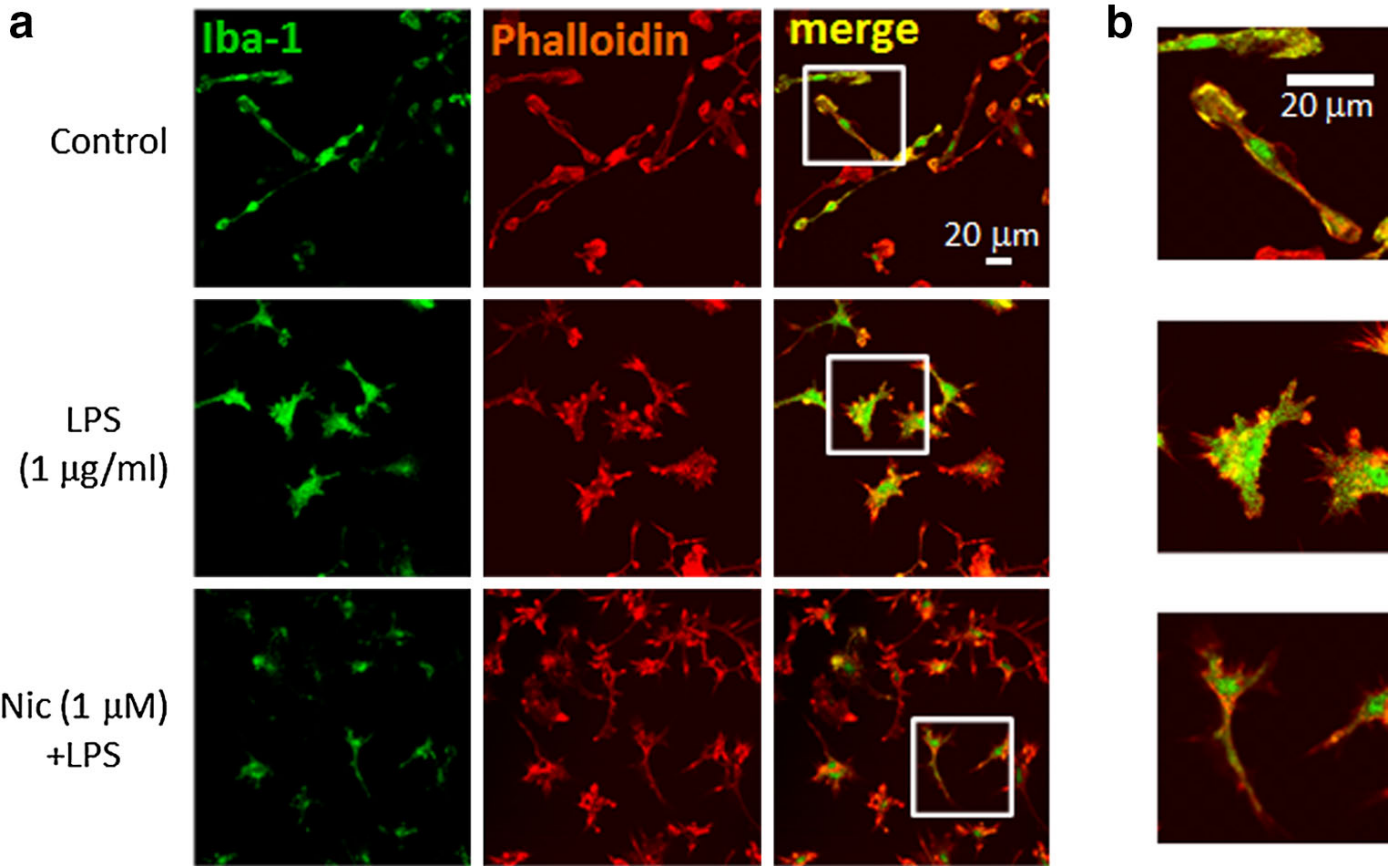
Effect of nicotine on LPS-induced morphological change of microglia. **a** Effects of LPS and nicotine on cellular morphology of microglia. Microglial cells were treated with LPS (1 μg/ml) for 24 h. Nicotine (1 μM) was pre-treated for 1 h before application of LPS. Immunofluorescence stained with anti-Iba-1 antibody (labeled with Alexa Fluor 488; *green*), and anti-phalloidin (anti-F-actin antibody) (labeled with Alexa Fluor 568; *red*) are shown. **b** Images in *white squares* in **a** are enlarged with different scale. More filopodia and membrane ruffling (actin polymerization) are shown in LPS-treated microglia

The morphological change of microglia was also observed. Application of LPS for 24 h caused so-called activated shape of microglia; bigger cell bodies with retracted processes. However, pre-treatment of microglial cells with nicotine prevented the morphological change of microglia (Fig. 4).

### The mechanism of inhibitory effects of nicotine on LPS-induced proton currents in microglia

**Fig. 5:**
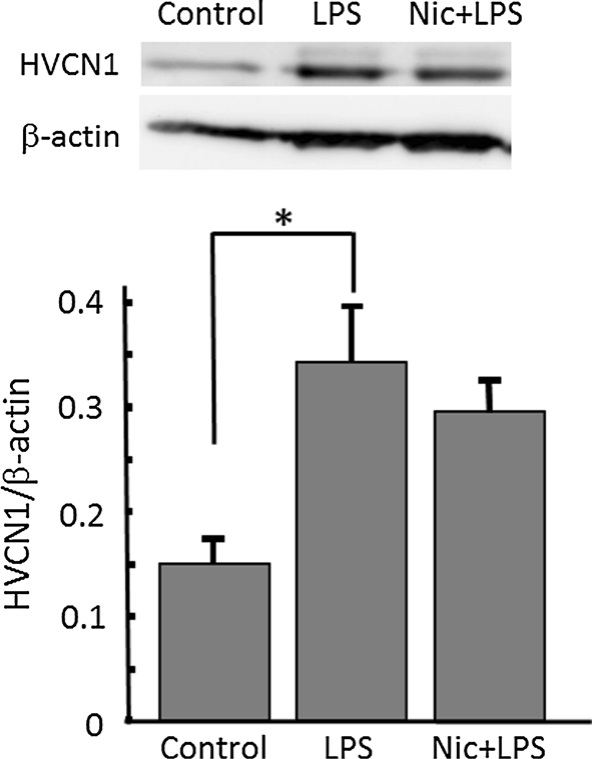
Nicotine does not affect LPS-increased expression of H^+^ channels in microglia. (*Upper panel*) Western blotting of H^+^ channel, HVCN1, and β-actin in cultured microglia. Microglial cells were treated with 1 μg/ml LPS for 24 h, and nicotine (1 μM) was pre-treated for 1 h before application of LPS. HVCN1 protein is detected at around 32 kDa in whole-cell lysate from microglia. (*Lower panel*) Relative expression levels of HVCN1 compared to β-actin are shown in control, LPS, and Nic + LPS. *p < 0.05 compared to control

First, the expression level of proton channel was tested by Western blotting. The expression of HVCN1 was significantly up-regulated by treatment of LPS (1 μg/ml) for 24 h. The pre-treatment with nicotine (1 μM) before application of LPS did not affect the up-regulated expression of HVCN1 (Fig. 5).

**Fig. 6:**
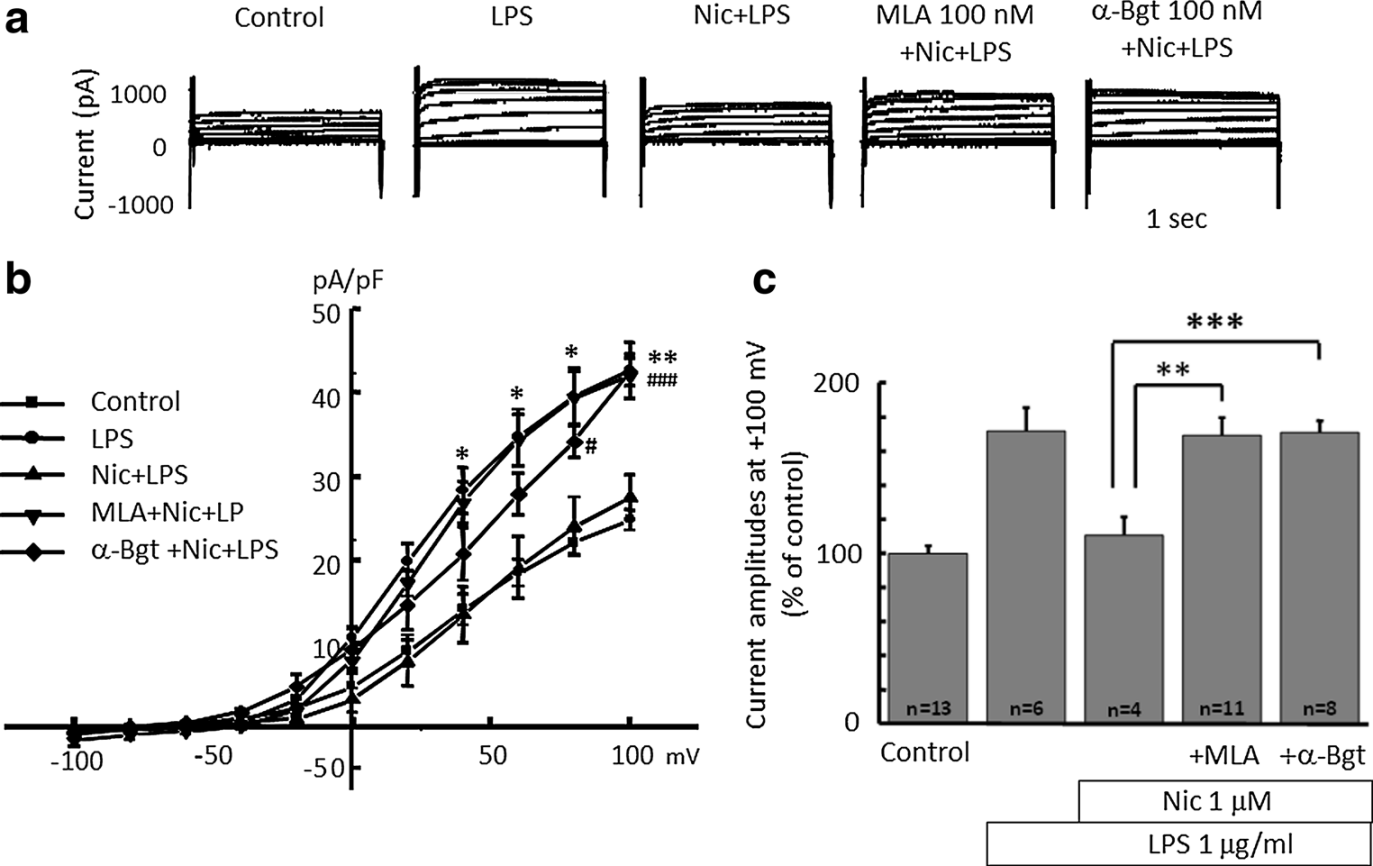
Nicotinic acetylcholine (nACh) receptor inhibitors cancel the effect of nicotine on LPS-increased proton current in microglia.** a** Current traces from −100 to +100 mV from the holding potential of −60 mV for 1 s are shown. Application of LPS (1 μg/ml) was for 24 h and nicotine (1 μM) was pre-treated for 1 h before application of LPS. Methyllycaconitine (MLA, 100 nM) and α-bungarotoxin (α-Bgt, 100 nM) were pre-treated for 30 min before application of nicotine, followed by LPS application. **b** I–V relationships in control (*filled square*), with 1 μg/ml LPS (*filled circle*), LPS with Nic (*filled upright triangle*), pre-treated with MLA (*filled downright triangle*) and α-Bgt (*open diamond*) are shown. **c** The relative current amplitudes at +100 mV in **b** are shown. **p < 0.01, ***p < 0.005 compared to LPS + Nic

Next, to test the involvement of the nicotinic acetylcholine receptor (nAChR), an α7 nAChR antagonist, methyllycaconitine (MLA) and α-bungarotoxin (α-Bgt) were applied. MLA (100 nM) or α-Bgt (100 nM) were applied 30 min before application of nicotine for 1 h, followed by application of LPS (1 μg/ml) for 24 h. The inhibitory effects of nicotine on LPS-induced H^+^ currents were cancelled by MLA or α-Bgt (Fig. 6).

### Nicotine inhibits neurotoxic effect of activated microglia

**Fig. 7:**
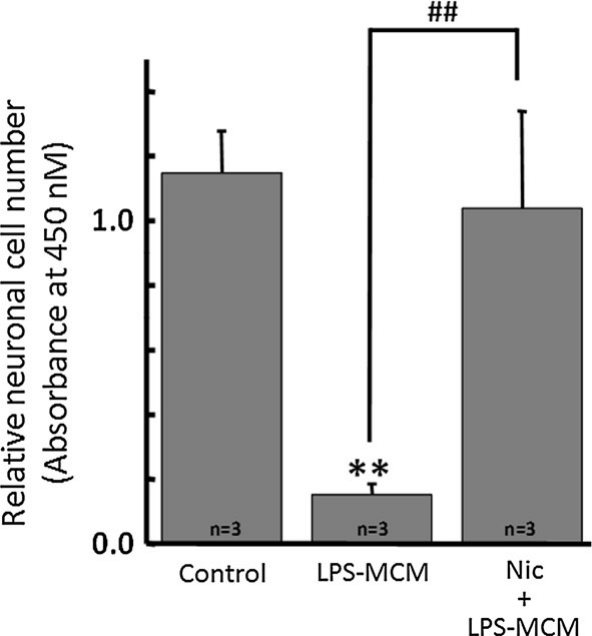
Nicotine inhibits neurotoxic effect of LPS-activated microglia. The conditioned medium from LPS-activated microglia (LPS–MCM) has neurotoxicity due to inflammatory cytokines. However, LPS–MCM from cells with nicotine (1 μM) pre-treatment significantly restored neuronal cells. **p < 0.01 compared to control. ^##^ *p* < 0.01 compared to LPS–MCM

LPS is known as a strong activator of microglia, leading production and release of pro-inflammatory cytokines and reactive oxygen species (ROS) [47–49]. To assess whether nicotine suppresses the LPS-mediated neurotoxic effect via microglia, LPS-treated microglial conditioned medium (LPS–MCM) with or without nicotine pre-treatment was applied to cultured neuronal cells. Due to LPS-induced inflammatory cytokines released from microglia, the number of living neuronal cell decreased significantly by application of LPS–MCM. However, LPS–MCM with pre-treatment of nicotine rescued neuronal cell damage, keeping the number of living cell intact (Fig. 7).

## Discussion

Our data suggest that nicotine inhibits H^+^ currents of microglia via interactions with α7 nAChRs. This means that α7 nAChRs agonists may have a therapeutic potential via regulation of microglial activation in neuroinflammatory diseases.

First, we showed that LPS activated H^+^ currents of microglia. An LPS-sensitive H^+^ current was previously reported in microglia [27–29, 50] and dendritic cells [51]. In our study, the electrophysiological recording was performed at 37 °C because the voltage-gated H^+^ channel was reported to be temperature sensitive [52].

As mononuclear phagocytic cells, microglial cells express high levels of superoxide-producing NADPH oxidases [53]. The sole function of members of the NADPH oxidase family is to generate reactive oxygen species (ROS) and upregulate the production of TNF-α [53, 54] that are believed to be important in CNS host defense [55]. However, ischemia can also lead to NADPH oxidase-induced ROS production and inflict damage on native cells. Therefore, the function of NADPH oxidases in microglia is a double-edged sword.

In our study, LPS-induced currents in microglia were completely inhibited by the NADPH oxidase inhibitor DPI (Fig. 2), suggesting that the currents were NADPH oxidase-dependent H^+^ currents. NADPH oxidase is electrogenic [56], generating electron current (Ie) [57, 58] with voltage-dependency [30]. Ie is compensated by H^+^ efflux mediated by voltage-gated H^+^ channels [59, 60], which may explain why phagocytes need H^+^ channels [30]. Voltage-gated H^+^ channels was required for NADPH oxidase-dependent ROS generation in brain microglia. Therefore, blocking either NADPH oxidase or H^+^ channels is useful to reduce neurotoxic effects due to activation of microglia and ROS generation.

Neutrophils exposed to LPS upregulates NDPH oxidase assembly [45]. Since nicotine inhibits fibrillar β amyloid peptide (1-42) (fAβ_1-42_)-induced NADPH oxidase activation [61], it is likely that nicotine inhibits LPS-induced NADPH oxidase activation as well.

The LPS-induced H^+^ currents of microglia were inhibited by nicotine (Fig. 3). Though LPS up-regulated expression of HVCN1, nicotine did not affect the LPS-increased expression of HVCN1 (Fig. 5). It is likely that nicotine affects the function of H^+^ channel, either single channel conductance or open probability, not the signal pathway on the way or during the transcription of H^+^ channel gene. This functional change of H^+^ channel should be investigated in the future. The inhibitory effect of nicotine was also observed morphologically in LPS-treated microglia (Fig. 4). The typical morphological change in LPS-treated microglia is retracting processes and becoming non-polar, assume a large, round, flat shape, and gradually develop many microspikes all over the cell body [49]. However, if the cells were pre-treated with nicotine, the morphological change was almost cancelled, suggesting the NADPH oxidase-H^+^ channel cascade is also involved in LPS-induced change in microglial morphology.

The question is how does nicotine inhibit the H^+^ current? To test whether nicotine affects the H^+^ channel directly or indirectly, we tested the involvement of α7 nAChR. Both α-Bgt and MLA, α7 nAChR antagonists, cancelled the inhibitory effect of nicotine on LPS-induced H^+^ current (Fig. 6). This means that down-stream signaling of the α7 nAChR mediates the inhibitory effect on NADPH-H^+^ channel cascade. Nicotine can alkalinize intracellular solution [62], reducing H^+^ concentration. This could reduce the outward H^+^-current. However, it is unlikely because nicotine concentration was quite low and the effect was blocked by α-Bgt and MLA.

The nicotine-induced Ca^2+^ signals, which are dependent on phospholipase C and inositol 1,4,5-trisphosphate (IP_3_), modulated the release of TNF-α in response to either activation of P2X_7_ receptors (positive modulation) or LPS (negative modulation) [63]. The cholinergic inhibition of LPS-induced TNF-α release from microglia is mediated by the inhibition of p38 mitogen-activated protein kinase (MAPK) and p44/42 [64]. On the other hand, anti-depressants and local anesthetics inhibit the voltage-gated H^+^ channels in microglial cell lines [38, 39]. In T cells, lidocaine down-regulates nuclear factor-κB signaling and inhibits cytokine production [65]. Therefore, it is speculated that inhibition of the H^+^ channel results in the inhibition of LPS-induced cytokine production, though the precise signal pathway is not clear.

**Fig. 8:**
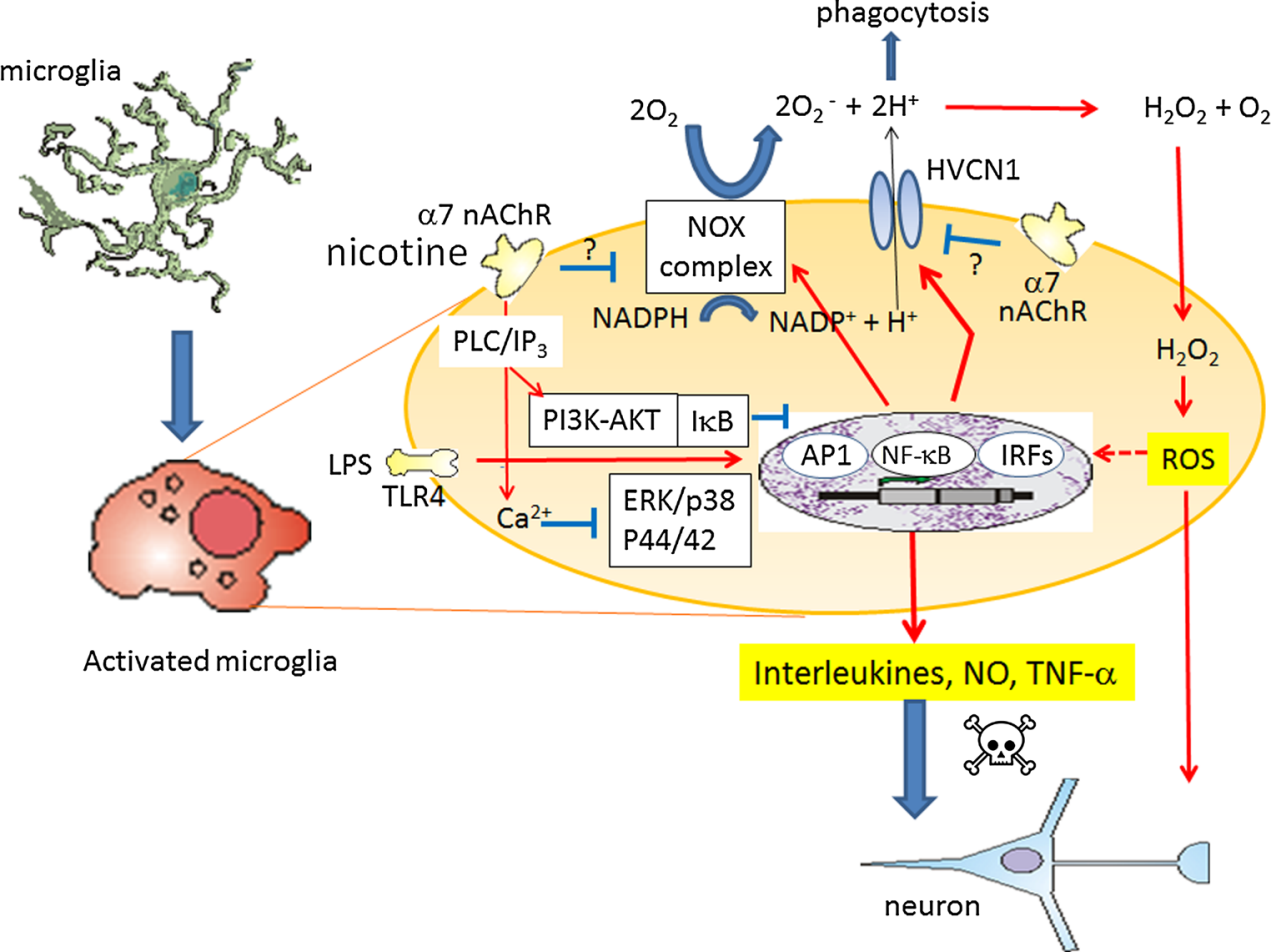
Proposed schema on inhibitory effects of nicotine on LPS-induced microglial activation. LPS, glycolipids found in the outer membrane of some types of Gram-negative bacteria, bind to Toll-like receptor 4 (TLR4) and activate signaling pathways; extracellular signal-regulated kinase (ERK)/p38 mitogen-activated protein kinase (MAPK), AP1, nuclear factor-κB (NF-κB), or IRFs (IRF3/IRF7), and hence production and release of pro-inflammatory cytokines, nitric oxide (NO) via inducible NO synthase and tumor necrosis factor-α (TNF-α). LPS also upregulates NADPH oxidase (NOX) assembly. The voltage-gated H^+^ channel, HVCN1, enables NOX function by compensating cellular loss of electrons with protons, which are required for phagocytosis. Furthermore, HVCN1 was required for NOX-dependent ROS generation. Nicotine binds to α7 nAChR in microglia, causing transient increase in intracellular Ca^2+^ in phospholipase C (PLC)/inositol 1,4,5-trisphosphate (IP3)-dependent manner [69], negatively modulates LPS-induced release of TNF-α. Cholinergic protection via α7 nAChR and PI3K-Akt pathway in LPS-induced neuroinflammation is also reported [70]. Nicotine may inhibit LPS-induced NOX. On the other hand, nicotine inhibits H^+^ current without affecting LPS-increased expression of HVCN1. Presumably, α7 nAChR signaling inhibits function of HVCN1 either directly or by inhibiting NOX, hence attenuating ROS production and further stimulation of pro-inflammatory cytokines, NO and TNF-α

Taken together, it is suggested that the inhibitory effect of nicotine on H^+^ current in LPS-stimulated microglia is mediated either by blocking NADPH oxidase or indirectly by α7 nACh signaling. Consequently, inhibiting H^+^ current reduces the production of ROS and subsequent formation of pro-inflammatory cytokines NO and TNF-α. The functional change of H^+^ channel and whether or not H^+^ channel is modified by down-stream signaling of α7 nAChRs should be investigated in the future (Fig. 8).

As for the beneficial effect of inhibition of H^+^ channel by nicotine, brain damage from ischemic stroke [26, 66, 67], or neurodegenerative disorders due to neuroinflammation [64, 68] were reported. As mentioned above, inhibition of the H^+^ channel would result in attenuation of the production of ROS, pro-inflammatory cytokines, and TNF-α. This many reflect why nicotine reduced LPS-induced neuronal cell death when they were co-cultured with microglia (Fig. 7) and therefore nicotine may have therapeutic effects on stroke or neurodegenerative disorders.

Further investigations on molecular signaling from activation of α7 nAChRs to inhibition of H^+^ currents in microglia will be needed. It is also important to investigate how LPS increases the expression of H^+^ channel. Anyhow, it is suggested that α7 nAChRs in microglia may have a therapeutic potential in neuroinflammatory diseases.
